# Self-discrepancies in mind perception for actual, ideal, and ought selves and partners

**DOI:** 10.1371/journal.pone.0295515

**Published:** 2023-12-13

**Authors:** Oliver L. Jacobs, Farid Pazhoohi, Alan Kingstone

**Affiliations:** 1 Department of Psychology, University of British Columbia, Vancouver, British Columbia, Canada; 2 School of Psychology, University of Plymouth, Plymouth, England, United Kingdom; University of Glasgow, UNITED KINGDOM

## Abstract

Defining and measuring self-discrepancies in mind perception between how an individual sees their actual self in comparison to their ideal or ought self has a long but challenging history in psychology. Here we present a new approach for measuring and operationalizing discrepancies of mind by employing the mind perception framework that has been applied successfully to a variety of other psychological constructs. Across two studies (N = 265, N = 205), participants were recruited online to fill in a modified version of the mind perception survey with questions pertaining to three domains (actual, ideal, ought) and two agents (self versus partner). The results revealed that participants idealized and thought they ought to have greater agency (the ability to do) and diminished experience (the ability to feel) for both themselves and their partner. Sex differences were also examined across both studies, and while minor differences emerged, the effects were not robust across the collective evidence from both studies. The overall findings suggest that the mind perception approach can be used to distill a large number of qualities of mind into meaningful facets for interpretation in relation to self-discrepancy theory. This method can breathe new life into the field with future investigations directed at understanding self-discrepancies in relation to prosocial behaviour and psychological well-being.

## Introduction

People perceive minds in other people, as well as other animals (e.g., cats and dogs), and even in nonbiological objects (e.g., robots). The field of work that is concerned with understanding these percepts is referred to as mind perception [1]. Mind perception has been applied to many different research areas within psychology and computer science [2]. However, surprisingly little, if any, work in mind perception has focused on investigating beliefs about one’s own mind.

There has, however, been a considerable amount of research following Rogers’ initial work linking psychotherapy outcomes with differences between domains of selves [3]. These included the real/actual self (the self that one actually is), the ideal self (the self that one desires to be), and the ought self (the self that one sees others as believing one should or ought to have). Roger’s work was later greatly expanded upon by Higgins’ landmark development of self-discrepancy theory which proposed that conflicting cognitive representations of the self result in emotional vulnerabilities and internal conflicts [4]. Indeed, differences between these three domains using a variety of scales [5,6] have been linked with depressed affect [7], suicidal ideation [8], and other specific affective states [9]. However, subsequent studies investigating these links have provided inconsistent results, in part due to how self-discrepancies have been operationalized [10,11] as well as psychometric properties of its assessments [12,13].

Notably, Higgins used self-questionnaires to measure self-discrepancies between actual, ideal, and ought selves. The Selves questionnaire developed over time [4,14] but was constructed by asking participants to ascribe adjectives and attributes for each of the categories of selves. Far from a perfect measure, this method highlights the difficulty in measuring discrepancies in qualities of mind, a problem that has plagued investigations into the subject even as additional domains of selves have been investigated [15]. A more recent study [6] presented a newer, more robust method for measuring self-discrepancies based on integrating idiographic (individual) and nomothetic (group-based) tools. While it has been a valuable tool for validating predictions of self-discrepancy theory, it too uses the same method of allowing participants to self-generate attributes. The advantage of this method is that the attributes that are important to the self are included. A fundamental disadvantage, however, is that direct comparisons between different attributes that vary between individuals are not possible.

In sum, the inconsistent operationalization of self-discrepancies and the questioning of its psychometric measures is at least in part a reflection of the difficulty in distilling qualities of mind into meaningful facets for interpretation. One potential remedy to this problem is to employ the mind perception framework developed by Gray et al. [16] which has been applied so successfully in many other contexts [17,18]. A major advantage of this approach to understanding how people perceive minds is that the majority of variance in people’s perceptions of mind can be distilled into two distinct principal factors. Gray et al.’s framework [16], derived from factor analysis, was the formation of a 2-factor model with one factor labeled experience (associated with capabilities related to feeling) and one factor labeled agency (associated with capabilities related to doing). In turn, the perception of these two factors, experience and agency, have been linked with meaningful predictors of real-world behaviour each in their unique way such as with personality [19], psychopathology [20], and moral attitudes [21].

In the original study by Gray et al. [16], one of the many targets in question were the participants themselves. In other words, participants were asked to rate themselves on a wide variety of capacities of mind. This in effect began to tap into metacognitive beliefs about one’s own mind or one’s actual self. Gray et al. found that people rated themselves higher on agency and experience than other animals, and they rated themselves higher again than a dead person, a robot, or inanimate objects. Beyond this initial foray, to our knowledge there has been no systematic study investigating perceptions and attitudes towards people’s own minds using the mind perception framework. This is important because, as previously noted, the scope of attributes encompassed by more traditional self-discrepancy measures of mind introduces variability based on individuals and specific contexts. This may mask meaningful relationships and contribute to the inconsistent pattern of findings in previous literature. The mind perception framework has the potential to aid the categorization of qualities of mind into a more precise and consistent measure for understanding self-discrepancies of mind.

It is worthwhile to highlight the subtle distinction between the self and the mind herein. The self typically refers to an individual’s identity, personal characteristics, values, and attitudes [22,23]. It contains both the entirety of the ’hardware’ and ’software’ that makes us who we are. The mind, however, is conceptually thought of as the ‘cognitive machinery’ that enables us to come to think, feel, and understand the world around us which is encompassed by the self [24,25]. In the context of self-discrepancy theory, there is a key distinction regarding self-discrepancies of the self, more broadly, versus the mind. The extant literature on self-discrepancies regarding physical features of the self as with body image is itself a large corpus of research relative to the focus on qualities of mind or mental features [26–28]. The focus on qualities of mind in self-discrepancy theory is the branch particularly troubled by the inconsistent operationalization and psychometric issues [10–13] and is the branch in which the mind perception framework can potentially provide immediate value.

The purpose of the present research is to examine—using the mind perception framework—how people attribute values across different domains (actual, ideal, ought) and agents (self versus partner). We do so by first conducting an exploratory study with only actual versus ideal domains before following up with a pre-registered study involving all three domains (self, ideal, ought). The inclusion of two agents reflects our intuition that people may be motivated to desire different attributes for themselves compared to a relationship partner, in line with some of Higgins’ original findings that different emotional vulnerabilities relate to different kinds of self-discrepancies [4]. Finally, we examined sex differences to determine to what extent, if any, this factor influences discrepancy perceptions as a number of sex differences have been observed in other types of self-discrepancies, such as with body image [29,30].

## Study 1

### Method

#### Participants

The number of participants was determined using an a priori power analysis using WebPower in R [31]. We sought to detect a medium-sized effect (*d* = .5) with a desired power of 90% for the lowest powered analysis which involved an interaction with sex. This led to a required sample size of 114 for each sex.

In total, there were 265 participants that took part in the survey (147 men and 118 women; M age: 40.71, SD age: 11.44), with 192 participants indicating that they were currently in a relationship. All participants took part using IP addresses from the United States and were sampled using Amazon Mechanical Turk (MTurk) and had approval rates of 99% on prior surveys. The distribution of participants reporting their highest educational attainment were as follows: 27.2% had a high school diploma, 10.6% had a post-secondary diploma, 44.9% had an undergraduate degree, and 17.3% had a postgraduate degree. This study was approved by the ethics board of the University of British Columbia (H10-00527) and all participants provided informed consent to participate.

### Material and procedure

A Qualtrics survey was distributed to participants using the crowd-sourcing program MTurk, which is a common tool for online data collection used in behavioural sciences [32]. After reviewing an ethics form and consenting to take part, participants were asked to fill in a basic demographic questionnaire with items pertaining to age, sex, and relationship status. Next, participants were given instructions that they would be asked to rate their current self, their ideal self, their current partner (if applicable), and their ideal partner on 6 questions on a 5-point numerical scale for each of the domains/agents (i.e. “For the following questions please answer them according to how you would assess your current self.”, “For the following questions please answer them according to how you would assess your ideal self.”, “For the following questions please rate your current partner as he or she currently is.”, and “For the following questions please imagine an ideal partner. This person would be ’the person of your dreams’.”). The 6 questions for each set of ratings were based on an updated version of the Mind Survey [16,19]. These were: ‘How capable of feeling fear are you?’, ‘How capable of exercising self-control are you?’, ‘How capable of feeling self-pleasure are you?’, ‘How capable of remembering are you?’, ‘How capable of feeling hunger are you?’, and ‘How capable of acting morally are you?’. For the sections asking participants to rate their ideal self these questions were changed from present to future tense specifically by changing ‘are you’ to ‘would you be’. Similar adjustments were made for the ratings of an ideal partner. The individuals who indicated they are currently in a relationship were presented with another block that included questions about their current partners, e.g., “How capable of remembering is your current partner?”. The item for self-pleasure as part of the experience factor was removed for improved internal consistency. The resulting Cronbach’s alphas for the self were: actual agency, a = .74, actual experience, a = .60 (formerly .59), ideal agency, a = .83, and ideal experience, a = .73 (formerly .53). The internal consistency for partners was as follows: actual agency, a = .74, actual experience, a = 64 (formerly .53), ideal agency, a = .76, and ideal experience, a = .72 (formerly .53). Participants were also asked an attention check question between the demographic and mind survey questions. After completing all questions, participants were thanked for participating and compensated for their time.

## Results and discussion

**Fig 1:**
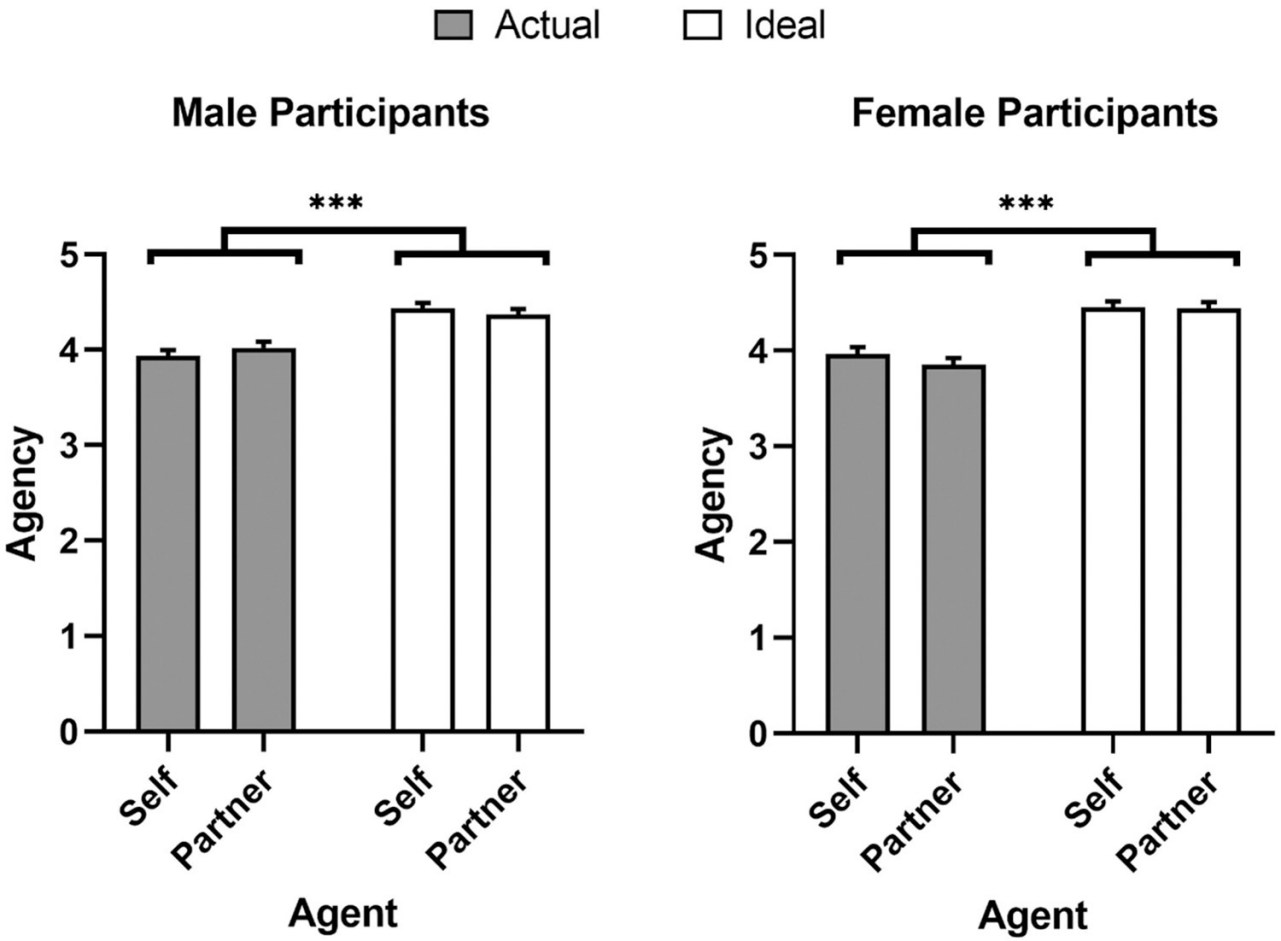
Mind perception ratings of agency. Means and SEM for agency ratings as a function of agent, domain, and participant sex. * *p* < 0.05, ** *p* < 0.01, *** *p* < 0.001.

A linear mixed model was conducted to investigate the effect of agent (self and partner), domain (actual and ideal), and participant sex (male and female) as fixed factors on agency ratings (Fig 1) while participant was included as a random factor. The main effect of domain was significant, *β* = 0.48, *SE* = 0.03, *df* = 723.76, *t* = 14.98, *p* < .001; individuals preferred higher agency for ideal agents (*M* = 4.42, *SEM* = 0.03, 95% CI [4.35, 4.50]) than actual ones (*M* = 3.95, *SEM* = 0.03, 95% CI [3.87, 4.02]. No other main effects nor any interactions were significant (all *p*’s > .05).

**Fig 2:**
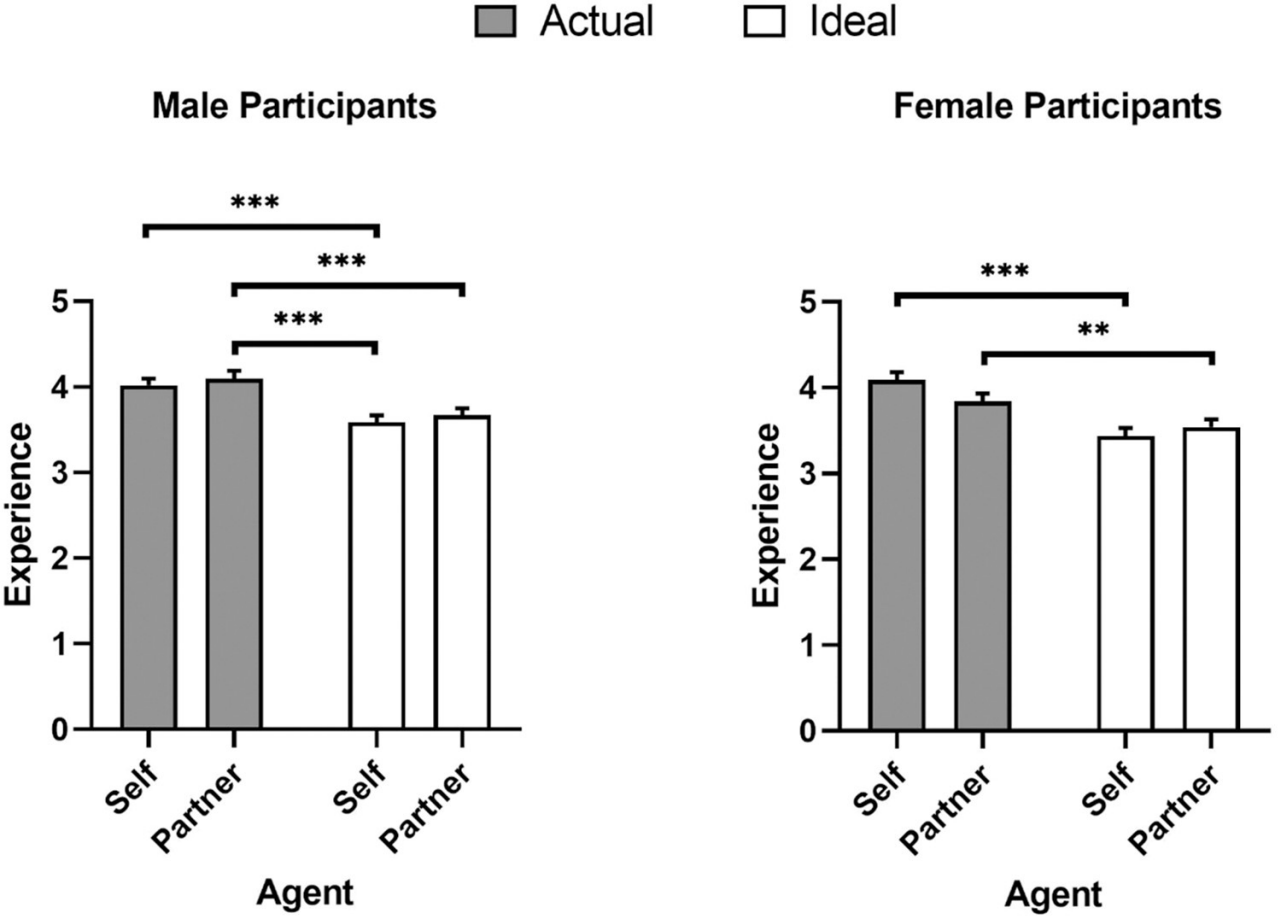
Mind perception ratings of experience. Means and SEM for experience ratings as a function of agent, domain, and participant sex. * *p* < 0.05, ** *p* < 0.01, *** *p* < 0.001.

Another linear mixed model was conducted predicting experience ratings with agent (self and partner), domain (actual and ideal) and participant sex (male and female) as fixed factors, in addition to participant being included as a random factor (Fig 2). The main effect of domain was significant, *β* = -0.45, *SE* = 0.04, *df* = 722.47, *t* = -11.89, *p* < .001. Moreover, significant two-way sex × agent and domain × agent interactions were qualified by a significant three-way agent × domain × sex interaction, *β* = 0.36, *SE* = 0.15, *df* = 722.47, *t* = 2.35, *p* = .019. Pairwise comparisons with Bonferroni corrections showed that men preferred less experience for their ideal self (*M* = 3.59, *SEM* = 0.08, 95% CI [3.44, 3.74], *p* < .001) and ideal partners (*M* = 3.67, *SEM* = 0.08, 95% CI [3.52, 3.82], *p* < .001) than their actual partners (*M* = 4.10, *SEM* = 0.09, 95% CI [3.93, 4.27]). Men also preferred less experience for their ideal self than their actual self (*M* = 4.02, *SEM* = 0.08, 95% CI [3.87, 4.17], *p* < .001). Similarly, women preferred less experience for their ideal self (*M* = 3.44, *SEM* = 0.09, 95% CI [3.27, 3.61]) than their actual self (*M* = 4.09, *SEM* = 0.09, 95% CI [3.92, 4.26], *p* < .001). Moreover, women preferred less experience for their ideal partner (*M* = 3.54, *SEM* = 0.09, 95% CI [3.37, 3.71]) than their actual partner (*M* = 3.84, *SEM* = 0.09, 95% CI [3.66, 4.02], *p* = .010) The main effects of agent and sex, and sex × domain interaction were not significant (all *p*’s > .228).

The results from Study 1 indicate that participants strongly differentiated between agency and experience. Men and women desired greater agency but reduced experience across agents being assessed (self or partner). This split across facets of mind perception reflect similar patterns found in other applications of mind perception that suggest each facet predicts unique clusters of real-world behaviour [19–21]. Finally men, unlike women, indicated that they perceived more experience in their current partners than ideal selves. See Table 1 for the means and standard deviations of each mind perception item.

**Table 1:** Means and standard deviations for mind perception factors.

			Experience		Agency		
			Fear	Hunger	Self-control	Remembering	Acting Morally
Self	Mean	Ideal	3.36	3.68	4.35	4.42	4.53
	SD		1.19	1.18	0.89	0.83	0.75
	Mean	Actual	3.91	4.19	3.76	3.83	4.28
	SD		1.05	0.97	1.01	0.96	0.76
Partner	Mean	Ideal	3.44	3.79	4.28	4.32	4.61
	SD		1.12	1.06	0.83	0.74	0.65
	Mean	Actual	3.81	4.22	3.77	3.83	4.29
	SD		1.02	0.89	1.01	0.97	0.78

## Study 2

Following the results of Study 1, an additional study became desirable to examine the robustness of the prior results through replication with a new sample. The secondary aim of Study 2 is to also include an additional domain, the ought self, for assessment. This study was pre-registered and is available for viewing on OSF: https://osf.io/gwhc6.

The hypotheses for Study 2 follow from the results of Study 1. We expect that individuals will desire greater agency and less experience in their ideal selves and ideal partners compared to their actual selves and actual partners. The new hypothesis concerns the addition of the ought self. Specifically, we expect that individuals will express that the ought self and ought partner should possess greater agency and less experience than the actual self and partner. This hypothesis stems from the idea that the ought self is heavily shaped by societal norms and expectations similar to perceptions of the ideal self. In this case, we believe that norms that value competence and leadership will lead to believing others think one ought to have greater agency, and norms about avoiding vulnerability will lead to believing others think one ought to have less experience.

## Method

### Participants

205 participants took part in the survey (94 men and 111 women; M age: 41.63, SD age: 10.97), with all participants indicating that they were currently married or in a relationship (a criterion for eligibility to take part). All participants took part using IP addresses from the United States and were sampled using Amazon Mechanical Turk (MTurk) and had approval rates of 99% on prior surveys. The distribution of participants reporting their highest educational attainment were as follows: 21.0% had a high school diploma, 8.8% had a post-secondary diploma, 44.9% had an undergraduate degree, one indicated elementary school and 24.8% had a postgraduate degree.

### Material and procedure

The materials and procedure closely resembled Study 1. After consenting to take part, participants were asked to indicate their age, sex, and education. Qualtrics was used for the dissemination of the survey through MTurk. After the demographic questions, participants were presented at random each of the domains (actual, ideal, ought) and agents being assessed (self vs. partner). The instructions were the same as in Study 1 (e.g., “For the following questions please answer them according to how you would assess your current self.”) with the exception being the novel instructions for the ought self (“For the following questions, please answer them according to how you would assess what others think you ought to be (should be like)” and ought partner (“For the following questions, consider the attributes that external parties—such as family, friends, or society at large—deem essential or desirable in your partner.”). The same 6 questions from the Mind survey [16,19] were asked for each of the domains and agents. Again, these were: ‘How capable of feeling fear are you?’, ‘How capable of exercising self-control are you?’, ‘How capable of feeling self-pleasure are you?’, ‘How capable of remembering are you?’, ‘How capable of feeling hunger are you?’, and ‘How capable of acting morally are you?’. These questions were again modified to grammatically fit the accompanying domain/agent being assessed. The item for self-pleasure as part of the experience factor was removed for improved internal consistency once again. The resulting Cronbach’s alphas for the self were: actual agency, a = .65, actual experience, a = .55 (formerly .59), ought agency = .76, ought experience = .64 (formerly .60), ideal agency, a = .77, and ideal experience, a = .67 (formerly .53). The internal consistency for partners was as follows: actual agency, a = .71, actual experience, a = .54 (formerly .56), ought agency = .72, ought experience = .65 (formerly .64), ideal agency, a = .80, and ideal experience, a = .61 (formerly .53). Participants were also asked an attention check question among the mind ratings. After completing all questions, participants were thanked for participating and compensated for their time.

### Results and discussion

**Fig 3:**
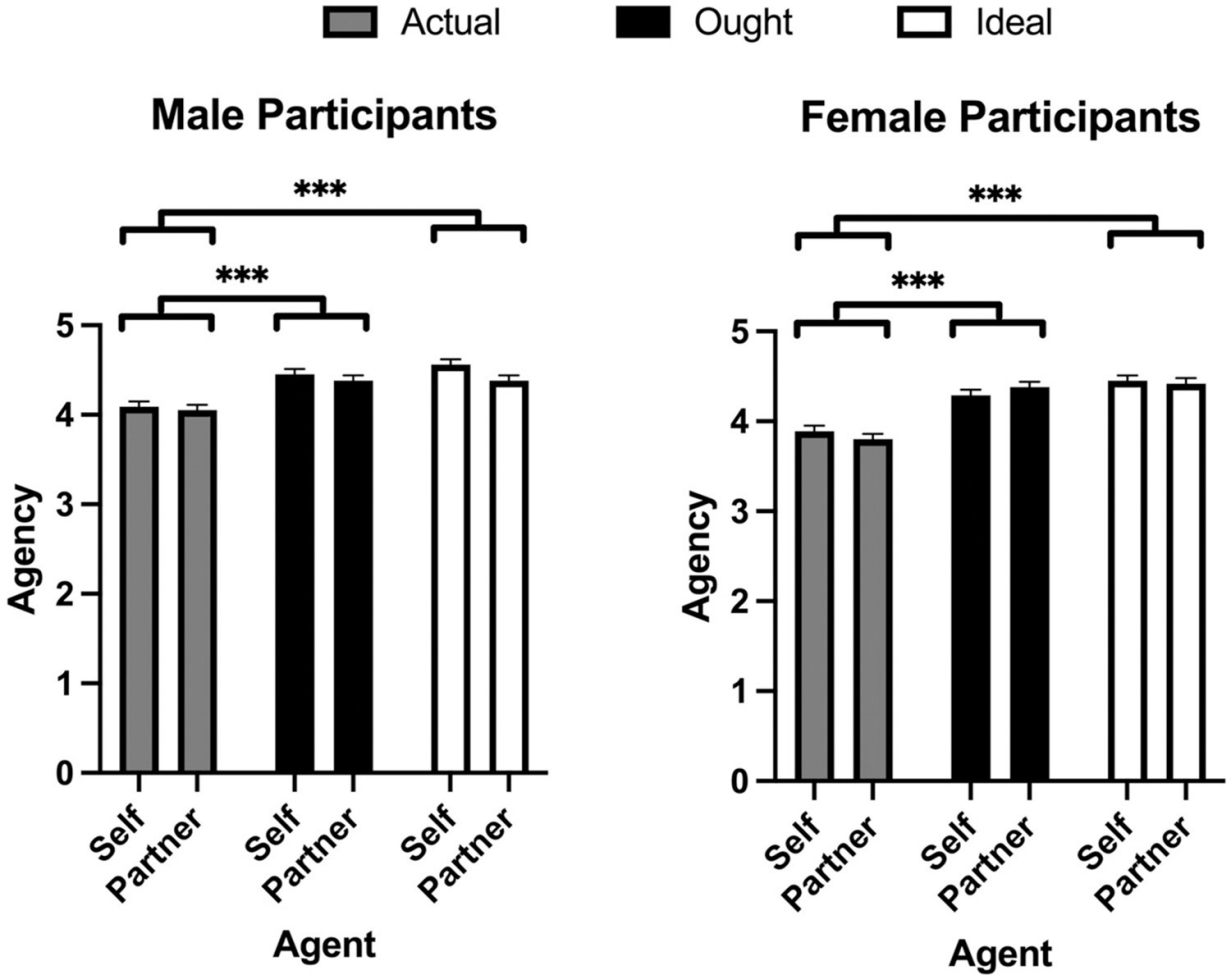
Mind perception ratings of agency in Study 2. Means and SEM for agency ratings as a function of agent, domain, and participant sex. * *p* < 0.05, ** *p* < 0.01, *** *p* < 0.001.

A linear mixed-effects model was used to examine the influence of domain (actual, ideal, ought), agent (self vs partner), and participant sex on agency ratings with participant as a random effect (Fig 3). The fixed effects omnibus test revealed a main effect of domain, *F*(2,1015) = 131.98, *p* < .001, a main effect of agent, *F*(1,1015) = 4.05, *p* = .044, and an interaction between participant sex and domain, *F*(2,1015) = 4.62, *p* = .010. The main effect of sex, the interaction between sex and agent, and the 3-way interaction were all nonsignificant; all *p*’s > 0.05. The fixed effects parameter estimate for agent revealed that across both sexes, people attributed greater agency to themselves (*M* = 4.29, *SEM* = 0.03, 95% CI [4.17, 4.31]) versus their partner, (*M* = 4.24, *SEM* = 0.03, 95% CI [4.22, 4.36], *t*(1015) = 2.011, *p* = 0.044). Moreover, post-hoc Bonferroni comparisons between domains revealed that participants rated the ideal domain (*M* = 4.46, *SEM* = 0.03, 95% CI [4.38, 4.53]) higher than the ought (*M* = 4.38, *SEM* = 0.03, 95% CI [4.30, 4.45], *p* = 0.046) and actual domains (*M* = 3.96, *SEM* = 0.03, 95% CI [3.88, 4.03], *p* < .001). They also rated the ought domain significantly higher than the actual domain (*p* < .001).

The post-hoc comparisons on the sex by domain interaction revealed that among women, participants desired greater agency for their ideal scores (*M* = 4.44, *SEM* = 0.05, 95% CI [4.34, 4.54]) compared to their actual scores (*M* = 3.83, *SEM* = 0.05, 95% CI [3.74, 3.95], *p* < .001), and greater agency for their ought scores (*M* = 4.34, *SEM* = 0.05, 95% CI [4.24, 4.44]) than actual scores, (*p* < .001). Among men, the same pattern occurred wherein actual scores (*M* = 4.07, *SEM* = 0.06, 95% CI [3.96, 4.18], were significantly lower than ideal (*M* = 4.47, *SEM* = 0.06, 95% CI [4.36, 4.58], *p* < .001) and ought scores (*M* = 4.41, *SEM* = 0.06, 95% CI [4.30, 4.52], *p* < .001). Between sexes, female actual scores were lower than male ideal (*p* < .001) and ought scores (*p* < .001). Male actual scores were likewise lower than female ideal (*p* < .001) and ought scores (*p* < .001). Finally, female actual scores were lower than male actual scores (*p* < .001).

These results replicate the main finding from Study 1, specifically, that across sexes, people desire greater agency in their ideal selves and partners compared to their actual selves and actual partners. Similarly, it showed that people desired greater agency for their ought selves and ought partners. The marginal differences between Study 1 and 2 are that both sexes rated their actual selves as having greater agency than their partners and that men rated their actual selves higher than women rated their actual selves.

**Fig 4:**
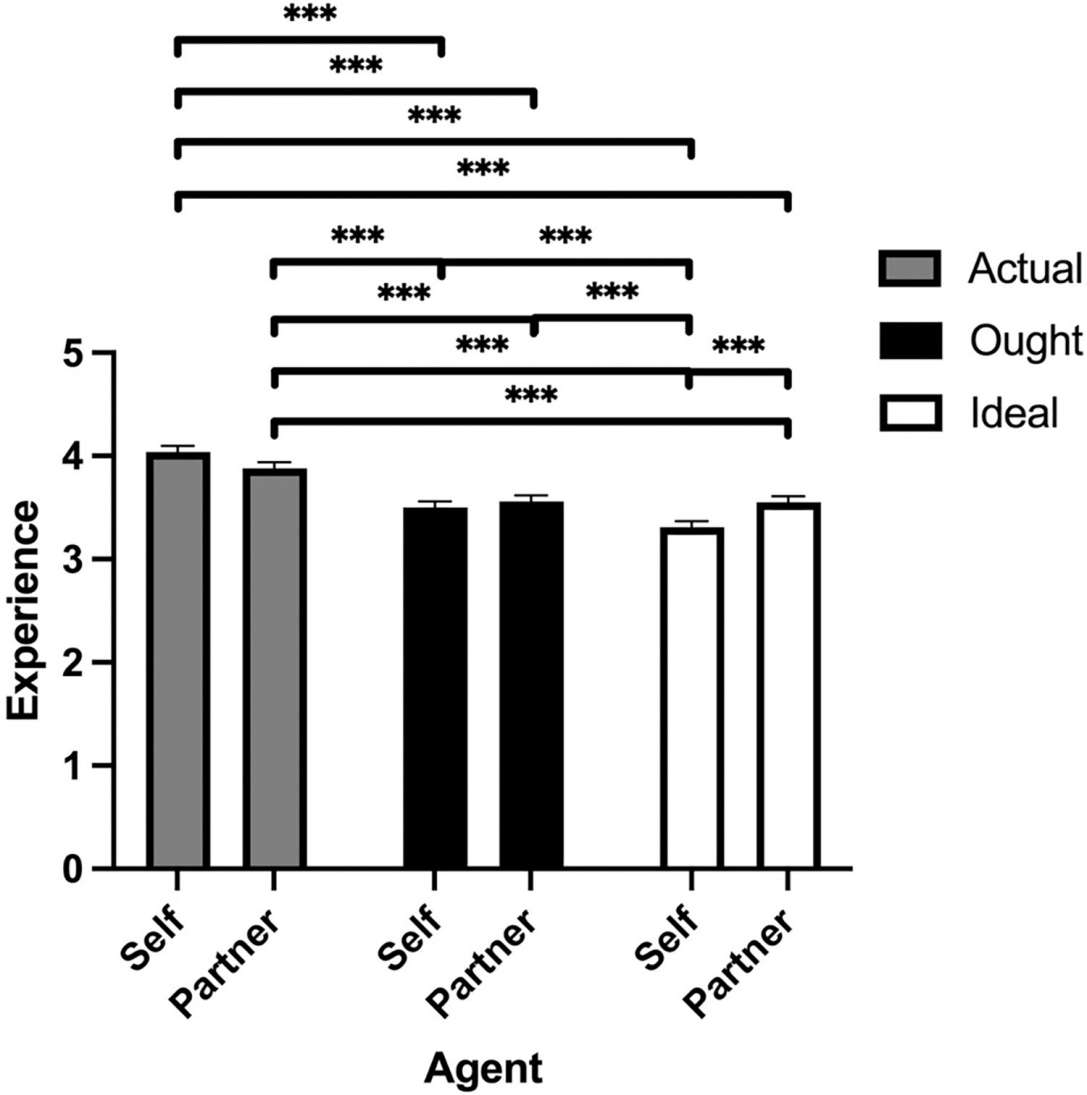
Mind perception ratings of experience in Study 2. Means and SEM for experience ratings of agent and domain. * *p* < 0.05, ** *p* < 0.01, *** *p* < 0.001.

For experience ratings, another linear mixed-effects model was used with domain (actual, ideal, ought), agent (self versus partner), and participant sex as fixed effects and participant as a random effect (Fig 4). The fixed effects omnibus test revealed a main effect of domain, *F*(2,1015) = 94.62, *p* < .001, and an interaction between domain and agent, *F*(2,1015) = 11.62, *p* < .001. Other main effects and interactions were all nonsignificant, *p*’s > .05.

Post-hoc comparisons of experience ratings between domains revealed that participants desired less experience in their ideal scores (*M* = 3.43, *SEM* = 0.05, 95% CI [3.32, 3.53]) than actual scores (*M* = 3.96, *SEM* = 0.05, 95% CI [3.58, 4.06], *p* < .001), ought scores (*M* = 3.53, *SEM* = 0.05, 95% CI [3.42, 3.63]) over actual scores (*p* < .001), and ideal scores over ought scores (*p* =  .038).

Post-hoc comparisons on the interaction between domain and agent were also examined. Participants rated their actual partner (*M* = 3.88, *SEM* = 0.06, 95% CI [3.76, 4.00]) as having more experience than their ideal partner (*M* = 3.55, *SEM* = 0.06, 95% CI [3.43, 3.67], *p* < .001), ideal self (*M* = 3.31, *SEM* = 0.06, 95% CI [3.19, 3.42], *p* < .001), ought partner (*M* = 3.56, *SEM* = 0.06, 95% CI [3.44, 3.68], *p* < .001), and ought self (*M* = 3.50, *SEM* = 0.06, 95% CI [3.38, 3.62], *p* < .001). Participants also rated their actual self (*M* = 4.04, *SEM* = 0.06, 95% CI [3.92, 4.16]) as having more experience than their ideal self (*p* < .001), ought self (*p* < .001), ideal partner (*p* < .001), and ought partner (*p* < .001). The remaining significant comparisons were that participants attributed more experience to their ideal partner than ideal self (*p* < .001), more experience to their ought self compared to ideal self (*p* = .015), and more experience to their ought partner than ideal self (*p* < .001).

In summary, the main results for experience attributions were very similar to Study 1. Participants attributed greater experience to their actual selves and partners than their ideal selves and ideal partners. Participants also attributed greater experience to their actual selves and partners than their ought selves and partners. These gaps between domains appeared larger for self-ratings than partner ratings. This latter finding could reflect that the desire for reduced vulnerability in oneself and partner is stronger for the self both ideally and for what people believe others think they ought to be like. See Table 2 for the means and standard deviations of each mind perception item.

**Table 2:** Means and standard deviations for mind perception factors.

			Experience		Agency		
			Fear	Hunger	Self-control	Remembering	Acting Morally
Self	Mean	Ideal	3.08	3.52	4.40	4.54	4.58
	SD		1.19	1.17	0.84	0.72	0.72
	Mean	Ought	3.33	3.65	4.30	4.29	4.50
	SD		1.10	1.08	0.84	0.75	0.71
	Mean	Actual	3.90	4.18	3.81	3.96	4.19
	SD		0.93	0.81	0.94	0.89	0.82
Partner	Mean	Ideal	3.37	3.72	4.35	4.37	4.49
	SD		1.01	0.99	0.78	0.72	0.72
	Mean	Ought	3.37	3.73	4.29	4.33	4.53
	SD		1.04	1.02	0.78	0.71	0.67
	Mean	Actual	3.63	4.12	3.79	3.74	4.21
	SD		1.03	0.84	0.96	0.93	0.76

## General discussion

Understanding the psychological consequences of self-discrepancies in domains of the self (i.e., actual, ideal, ought) has been a long, meritorious scientific endeavor. However, discrepancies regarding physical features (i.e. body image) are far better understood than discrepancies in mental qualities. In other words, while the field knows much about the causes and consequences of self-discrepancies between one’s actual, ideal, and ought body image [26–30], research into discrepancies in attributes of mind remain a much less investigated and a far more convoluted issue [12,13]. As noted in the introduction, we take as a working hypothesis that a key reason for the latter situation stems from the complexity in past measurements of mind that could introduce variability and restrict comparisons between individuals. Thus, we took a novel approach to this issue by using a 2-factor model of mind perception [16] to probe discrepancies in mind. A critical advantage of this approach is that it provides a succinct and common metric to both measure and interpret a variety of qualities of mind between individuals.

In Study 1, 265 participants took part in a MTurk distributed mind survey probing participants on their attitudes towards their current mind and their preferences for an ideal mind for themselves and their partners. The results clearly demonstrated that both men and women desire greater agency for themselves and their partners. In contrast, men and women want less experience for themselves and their partners while men, unlike women, also prefer less experience than their current partner.

In Study 2, a new sample of 205 participants were recruited using the same methodology as Study 1 but with the inclusion of an additional domain of the self—the ought self. The large discrepancies between actual and ideal ratings for one’s self and partner emerged again, and ratings for the ought self and ought partner displayed a similar pattern wherein people thought they ought to have more agency but less experience. In Study 2, participants also indicated they viewed themselves as having more agency than their partner regardless of their sex, and that men compared to women rated themselves higher on agency. Participants also stated they preferred less experience than their ideal partners, ought partners, and ought selves.

Across both studies the findings suggest several key ideas. First, mind perception frameworks can be used to elucidate discrepancies between domains of the selves similar to other methods that have been used to examine discrepancies in the past [6,9]. Additionally, and more importantly, the 2-factor model of mind perception reveals a distinction between the factors of agency and experience in terms of how people conceptualize their actual versus ideal or ought attributes of mind. People desire more agency in their ideal and ought self along with their partner. It is perhaps intuitive to discover that people desire greater self-agency and that they ought to have more of it. After all, people often lament their inability to do more or to do better. More surprising, is that our results on ratings of experience indicate that people wish and believe they ought to feel less. It would be unusual to hear remarks about wanting to feel less hope or sexual drive, or other forms of feeling—except perhaps for feelings of pain and depression. This finding that people idealize less experience is especially interesting given the fact that between agency and experience, Gray et al. [16] found experience to be the more important factor in terms of its ability to capture what makes us uniquely human.

The difference between agent (self versus partner) attributions in Study 2 also yielded an interesting pattern of results. The difference between one’s actual experience and ideal or ought experience is larger than those perceived in one’s partner. In part, this is due to the lower actual perceived experience in one’s self in addition to the higher levels of ideal or ought experience in one’s partner. If perceived experience is viewed as a form of moral patiency, as has been previously described [16], these findings suggest that people view themselves as somewhat more vulnerable than their partners, while ideally, they ought to be less. This fits a narrative that people believe they ought to be supportive and protective of their partners but want to avoid being the vulnerable partner themself.

The attitudes toward experience ratings and their association with moral patiency also may explain a more intriguing finding from Study 2. People believed they ought to have less experience than they do in reality, and they ideally would have even less than what they believe they ought to have. In essence, people ideally want to avoid being the target of moral right or wrong more than they believe others think it is acceptable to be. Perhaps this is reflecting an avoidance of victim blaming while considering a strong desire to avoid becoming a victim oneself.

In addition to the main findings, there were some intriguing sex differences between men and women. In Study 1, men preferred less sensitivity (i.e., experience) in their idealized self compared to their actual partners, whereas women did not. This finding did not re-emerge in Study 2. Similarly, a key sex difference in Study 2—that men rated their actual agency higher relative to women—was not found in Study 1. It could be that popular conceptualizations of masculinity being associated with less overt displays of emotion [33,34] may play a part in the sex effects for experience and agency. However, the collective evidence between experiments does not provide strong evidence that large sex differences exist in self-discrepancies between domains and agents—at least when examined within the mind perception framework—and thus the sex differences we observed are best treated with caution.

### Future directions and limitations

As an initial pair of studies delving into self-discrepancies between actual, ideal, and ought minds using a mind perception framework, these findings raise many questions for future research. For example, researchers may ask how people consider the minds of family members or friends, compared to strangers or foes? Or in a broader sense, how might individuals attribute mind to ingroup versus outgroup members? Mind perception has already been linked and discussed within the context of moral judgments [21,35]. Dehumanization, which involves the denial of universally human attributes [36], could map on to larger discrepancies between actual and ideal minds in particular outgroups. In other words, the mind perception approach that was applied here could be used to investigate whether dehumanizing percepts in part stem from larger discrepancies between actual and ideal minds reflected in the moral judgments of others. Furthermore, the denial of quintessentially human attributes suggests that self-discrepancies related to dehumanization would be particularly large on the experience factor of mind perception.

It is also worth noting that the present study had limitations and raises questions warranting further inquiry. The internal consistency of the mind perception survey had acceptable reliability only after the removal of the self-pleasure item for the experience factor across both studies, and in general, suggests that modifications to the survey may be required to probe self-discrepancies in mind for other purposes. More notably, the divergence of ideal qualities for agency and experience highlighted that there may be differences in the social desirability and valence of items in the mind perception survey. There appears to be more positive associations with agency items than experience items; a point surprisingly absent in the mind perception literature. One potential remedy would be to create new questions matched for valence (e.g. switching fear to courage or hunger to satiety) in future work applying the mind perception framework to self-discrepancies. Recent work has also revisited the optimal number of dimensions of mind perception with some advocating for a unidimensional factor structure [37–39], some advocating 3 factors [40,41] and some even advocating for 5 factors [42]. Interestingly, Malle [42] found that a 5-factor approach worked best when examining mind perception in desired robots–perhaps indicating that larger factor solutions may be optimal for examining idealized qualities of mind which future work could extend to humans in a method similar to the present approach.

In conclusion, the present work suggests that self-discrepancies between actual, ideal, and ought attributes of mind can be succinctly distilled using the mind perception framework. Using this framework, it was discovered that people in general idealize and believe they ought to have greater agency and weaker experiential qualities both for themselves and their partners. These findings suggest combining mind perception and self-discrepancy theory can provide new ways of investigating psychological well-being and prosocial behaviours such as moral judgments.

## Supporting information

S1 Checklist (DOCX)
